# The time has come for national clinical practice guidelines for managing late effects after cancer and cancer treatment

**DOI:** 10.2340/1651-226X.2024.40787

**Published:** 2024-06-23

**Authors:** Robert Zachariae, Peer Christiansen, Ali Amidi, Lisa Wu, Lise Ventzel, Nina Tauber, Annika von Heymann, Bolette Skjødt Rafn, Janne Fassov, Therese Juul, Peter Christensen, Christoffer Johansen

**Affiliations:** a Unit for Psychooncology and Health Psychology, Department of Oncology, Aarhus University Hospital, Aarhus, Denmark; b Department of Psychology and Behavioural Sciences, Aarhus University, Aarhus, Denmark; c Danish Breast Cancer Group Center for Late Effects (DCCL), Aarhus University Hospital, Aarhus, Denmark; d Department of Plastic and Breast Surgery, Aarhus University Hospital, Aarhus, Denmark; e Department of Oncology, Lillebaelt Hospital, University Hospital of Southern Denmark, Vejle, Denmark; f Center for Cancer Late Effects (CASTLE), Rigshospitalet, University of Copenhagen, Copenhagen, Denmark; g Danish Cancer Society Centre for Research on Survivorship and Late Adverse Effects After Cancer in the Pelvic Organs, Department of Surgery, Aarhus University Hospital, Aarhus, Denmark

**Keywords:** Survivorship, Clinical practice guidelines, management of late effects, symptom clusters, stepped care

The increasingly successful rates of cancer treatment, unfortunately, come at a considerable price in the form of late effects. The increasing incidence of most major cancer types and the growing number of cancer survivors underscore the need for clinical guidelines for managing both the organ- and treatment-specific late effects and the more general late effects occurring across cancer types and treatments. As we progress from understanding the basic building blocks of cancer to the intricacies of ongoing treatment advancement, we find ourselves in a critical juncture where we must determine how to effectively manage the costs of the remarkable survival success, ensuring comprehensive screening for and treatment of the many physiological, psychological, and social consequences that commonly affect people during and after cancer treatment.

International and national organizations such as the European Society of Medical Oncologists (ESMO), the National Comprehensive Cancer Network (NCCN), and the American Society of Clinical Oncologists (ASCO) have published clinical guidelines both for survivorship in general, e.g., [1, 2], and for the management of specific symptoms and late effects, e.g., anxiety and depression [3], insomnia [4], and fatigue [5]. However, to support the implementation of the best evidence-based approaches to manage late effects, there is also a need for national guidelines supporting the integration of the international initiatives at the national level.

Three national centers for research in late effects after cancer funded by the Danish Cancer Society^1^ have therefore taken the initiative to develop a set of national clinical practice guidelines for managing the most prevalent general cancer late effects across cancer types and treatments experienced by cancer survivors in the Danish setting. All authors of the present editorial are collaborating on developing guidelines for cancer-related depression, anxiety, sleep disturbance, fatigue, cognitive impairment, and pain.

Depression is significantly more prevalent among cancer survivors compared to the general population. Research shows that about 25% of people treated for cancer experience depression, which is markedly higher than the prevalence in the general population, where it varies around 5–10% [6]. For patients with moderate to severe symptoms of depression, the treatments recommended by international guidelines include cognitive behavioral therapy (CBT), behavioral activation (BA), and mindfulness-based therapy (MBT) [3].

Other distress-related issues are more specifically related to cancer, e.g., fear of cancer recurrence (FCR), where, depending on the cut-off used, between 58 and 20% of cancer survivors report clinically significant levels up to many years after completing primary treatment [7]. For FCR, the available evidence appears to favor so-called third-wave cognitive behavioral approaches, including metacognitive-based therapies, over traditional CBT [8].

While chronic insomnia is the most common sleep disturbance experienced by approximately 10% of the general population, it is, on average, three times more prevalent in cancer survivors [9]. As is the case in the existing guidelines for insomnia in the general population, international guidelines for managing insomnia in cancer survivors [4] recommend cognitive behavioral therapy for insomnia (CBTI) as first-line treatment. In contrast, the limited evidence for hypnotics does not outweigh the side effects and risks of adverse events associated with long-term use.

Cancer-related fatigue (CRF) is among the most prevalent and debilitating consequences of cancer and cancer treatments, with approximately one-third of cancer survivors experiencing clinically relevant levels of CRF up to 6 years post-treatment [10]. Existing cancer guidelines have evaluated and included various interventions, e.g., physical activity, energy conservation, light therapy, and psychosocial interventions [5]. While pharmacological treatment with stimulants has been considered, the burden of side effects may outweigh the benefits, and the available limited evidence indicates that non-pharmacological interventions, such as exercise and psychosocial interventions, alone or in combination, currently represent the best options [11].

Cancer-related cognitive impairment (CRCI) is another commonly reported late effect of cancer and its treatment, which has been reported in a broad range of non-CNS cancer populations across the cancer trajectory [12]. While often referred to by patients as ‘chemo-brain’, CRCI may occur as a result of both disease- and treatment-related factors. Although no standard treatment is available, evidence points to the beneficial effects of cognitive rehabilitation approaches [13] and physical activity [14].

Many cancer survivors experience pain, even many years post-treatment. For example, in a prospective study of colorectal cancer patients treated with adjuvant oxaliplatin, 21% had neuropathic pain in their feet 5 years after treatment [15]. Another study showed that neuropathic pain following surgery and chemotherapy represents a considerable burden to breast cancer survivors [16]. Neuropathic pain, defined as pain caused by a lesion or disease of the somatosensory nervous system, can be a result of a variety of treatments, including surgery, radiation therapy, chemotherapy, and other systemic treatments [17]. The national Danish guideline will focus on both pharmacological and non-pharmacological modalities for managing neuropathic pain [18, 19].

When developing guidelines, a specific challenge is that late effects after cancer treatment often manifest as clusters of symptoms that vary depending on factors like the type of cancer, treatment received, and individual’s health [20]. For example, in a longitudinal study of late effects in breast cancer survivors, one in five survivors was likely to be a member of a high-burden trajectory across all tested symptoms [21]. These symptom clusters may emerge and persist months to years after treatment, with the individual symptoms maintaining and exacerbating each other in a complex manner, affecting various aspects of physical and emotional functioning and well-being, requiring comprehensive assessment and management by healthcare professionals [22].

Across the different general late effects, it is generally recommended to use a stepped-care model, i.e., a structured method of delivering healthcare services where treatments are ‘stepped up’ (intensified) or ‘stepped down’ based on the individual’s needs and responses to treatment [23]. The efficacy of stepped-care approaches has increasingly been the focus of the investigation for several of the general late effects after cancer, e.g., FCR [24] and insomnia [25]. A stepped-care approach focuses on matching the level of care to the individual’s needs, starting with low-intensity options like self-help or community support and progressing to higher-intensity options such as therapy or medication if the initial interventions are insufficient. This approach aims to optimize resources, minimize unnecessary treatment, and ensure that individuals receive the most effective care for their specific needs.

The first step is to ensure that cancer survivors are screened at relevant intervals for late effects throughout their survivorship trajectory and that survivors presenting clusters of severe late effects receive a qualified assessment by a multidisciplinary team of clinicians with appropriate areas of expertise, including oncologists, surgeons, psycho-oncologists, and physiotherapists. One of the research centers (DCCL) has developed a digital tool focusing on early detection of late effects and offering guidance to both survivors and clinicians. Both DCCL and the Center for Late Effects After Cancer in the Pelvic Organs have established multidisciplinary videoconferences for assessing these late effects.

For most survivors, the second step may include digitally delivered information on late effects and educational strategies supporting survivors in preventive self-management strategies. For survivors with minor symptoms, physical after-treatment follow-up is likely to change its focus from possible cancer recurrence only to new responsibilities, including patient education and brief low-intensity interventions, e.g., delivered by nurses rather than oncologists [26] or as part of municipal cancer rehabilitation efforts.

The third step, among the least burdensome and most cost-effective options for survivors with single moderately severe symptoms, may be one of the growing numbers of digitally delivered interventions developed in recent years. For example, digitally delivered CBTI has been shown to be highly efficacious in treating insomnia in cancer survivors with derivative beneficial effects on fatigue [27].

Finally, at the fourth step, for the remaining group of survivors with clusters of several severe and complex late effects, there is a need for qualified, multidisciplinary assessment of and referral to specialized behavioral and physical interventions. This step also includes diagnosing and referring survivors with organ-specific late effects to relevant specialized clinics. For example, the Centre for Research on Survivorship and Late Adverse Effects After Cancer in the Pelvic Organs has developed clinical guidelines for late effects after cancers in the pelvic organs, e.g., colorectal cancer [28] and has treated more than 1,300 patients in specialized late effects clinics [29, 30].

National guidelines should thus not only provide recommendations for managing the individual late effects but also consider the total symptom load of survivors while ensuring the minimally intensive treatments corresponding to their needs. To patients, national guidelines represent standards and recommendations that can be referenced during consultations. Under certain circumstances, the existence of comprehensive national guidelines provides patients with the opportunity to assert their right to have specific symptoms assessed by clinicians. The entitlements to medical evaluation when presenting symptoms and receiving treatment and follow-up are fundamentally embedded in the contract between governments and citizens in many countries. The content of this contract undergoes constant evaluation, critique, and development. The call for national guidelines in late effect diagnostics and the provision of treatment algorithms in cancer treatment reflect this ongoing evolution.

## Footnotes

1 The three Research centers are: a) Danish Breast Cancer Group Center for Late effects (DCCL), Aarhus University Hospital, Aarhus, Denmark, b) Danish Cancer Society Centre for Research on Survivorship and Late Adverse Effects After Cancer in the Pelvic Organs, Aarhus University Hospital, Aarhus, Denmark, and c) Center for Cancer Late Effects (CASTLE), Rigshospitalet, University of Copenhagen, Copenhagen, Denmark
